# The *Shigella* Type III Secretion Effector IpaH4.5 Targets NLRP3 to Activate Inflammasome Signaling

**DOI:** 10.3389/fcimb.2020.511798

**Published:** 2020-09-30

**Authors:** Xiaolin Wang, Jin Sun, Luming Wan, Xiaopan Yang, Haotian Lin, Yanhong Zhang, Xiang He, Hui Zhong, Kai Guan, Min Min, Zhenxue Sun, Xiaoli Yang, Bin Wang, Mingxin Dong, Congwen Wei

**Affiliations:** ^1^Basic Medical College, Qingdao University, Qingdao, China; ^2^Department of Genetic Engineering, Beijing Institute of Biotechnology, Beijing, China; ^3^Department of Gastroenterology and Hepatology, The Fifth Medical Center of Chinese PLA (People's Liberation Army) General Hospital, Beijing, China; ^4^Third Medical Center of Chinese PLA (People's Liberation Army) General Hospital, Beijing, China

**Keywords:** *Shigella*, IpaH4.5, NLRP3, inflammasome, T3SS

## Abstract

Activation of the NLRP3 inflammasome requires the expression of NLRP3, which is strictly regulated by its capacity to directly recognize microbial-derived substances. Even though the involvement of caspase-1 activation in macrophages *via* NLRP3 and NLRC4 has been discovered, the accurate mechanisms by which *Shigella* infection triggers NLRP3 activation remain inadequately understood. Here, we demonstrate that IpaH4.5, a *Shigella* T3SS effector, triggers inflammasome activation by regulating NLRP3 expression through the E3 ubiquitin ligase activity of IpaH4.5. First, we found that IpaH4.5 interacted with NLRP3. As a result, IpaH4.5 modulated NLRP3 protein stability and inflammasome activation. Bacteria lacking IpaH4.5 had dramatically reduced ability to induce pyroptosis. Our results identify a previously unrecognized target of IpaH4.5 in the regulation of inflammasome signaling and clarify the molecular basis for the cytosolic response to the T3SS effector.

## Introduction

Innate immunity is the first barrier of the body against the invasion of pathogenic microorganisms. As an important component of the innate immune system, innate immune cells recognize pathogen-associated molecular patterns (PAMPs) through pattern recognition receptors (PRRs), followed by secreting cytokines and antimicrobial intermediates (Thompson et al., 2011). PRRs are sensors that detect typical molecules of pathogens and play key roles in defending against microbial invasion; they can be divided into membrane-bound PRRs and cytoplasmic PRRs (Fukata et al., 2009; Prajapati et al., 2014). Among all kinds of PRRs, nucleotide-binding oligomerization domain (NOD)-like receptors (NLRs) are cytoplasmic proteins that recognize specific PAMPs and activate pro-inflammatory and antimicrobial immune responses (Franchi et al., 2008; Caruso et al., 2014). NLRs consist of C-terminal leucine rich repeats (LRRs), a central nucleotide-binding oligomerization domain (NOD or NACHT), and an N-terminal domain. LRRs sense PAMPs, the NOD domain has intrinsic ATPase activity and the N-terminal domain mediates protein-protein interactions (Hamada et al., 2013; Dadi et al., 2018). Importantly, some NLRs, including NLRP1 (NLR-family PYD domain-containing 1), NLRP3, NLRP6, and NLRC4 (NLR family CARD domain-containing 4), also function in the form of inflammasomes (Ye et al., 2008; Jamilloux et al., 2013). The most fully characterized inflammasome is the NLRP3 (also known as NALP3 or cryopyrin) inflammasome, which can be triggered by a variety of stimuli (Kanneganti et al., 2007; Yazdi et al., 2010; Zuliani-Alvarez et al., 2017). Typical activation of the NLRP3 inflammasome can be described as follows: in response to PAMP recognition, NLRs bind to adaptor apoptosis-associated speck-like protein (ASC) by PYD-PYD interactions, which form the NLRP3 inflammasome, recruiting pro-caspase-1, and activating caspase-1 (Maharana et al., 2017; Taghavi et al., 2017). Afterwards, caspase-1 induces IL-1β production and causes a series of cellular events such as pyroptosis (Srinivasula et al., 2002).

*Shigella* are common pathogenic bacteria that can invade human macrophages, dendritic cells, and epithelial cells, leading to severe diarrhea because of inflammatory responses caused enteritis (Watarai et al., 1996). As gram-negative bacteria, *Shigella* utilize a type III secretion (T3S) apparatus to inject virulence factors into host cells; One class of effector proteins named invasion plasmid antigen H (IpaH) is important for *Shigell*a pathogenicity and possesses 12 genes includes IpaH1.4, IpaH2.5, IpaH4.5, IpaH7.8, IpaH9.8, etc, which are encoded on the 220 kb virulence plasmid or chromosome of *Shigella* (Buttner et al., 2008; Ashida and Sasakawa, 2015). The IpaH family has a highly conserved N-terminal leucine-rich repeat domain and a highly conserved C-terminal region (Vourc'h et al., 2003). The crystal structures and subsequent study demonstrated that the C-terminal region of IpaH proteins is a new family of E3 ubiquitin ligases (Rohde et al., 2007; Takagi et al., 2016). Previous studies indicated that each of the IpaH family effectors has different pathogenic mechanisms (Menard et al., 1993). For instance, IpaH9.8 was reported to downregulate the NF-κB-mediated inflammatory response by targeting essential modifier (NEMO)/IκB kinase gamma (IKKγ) in BMMs, while IpaH7.8 targets glomulin and IpaH4.5 interacts with NF-kB p65 protein in macrophages and epithelial cells (Ashida et al., 2010; Wang et al., 2013; Suzuki et al., 2014). IpaH4.5 also targets RPN13 in the 26S proteasome to dampen cytotoxic T lymphocyte activation in macrophages and epithelial cells (Otsubo and Mimuro, 2019). A recent paper revealed the role of IpaH4.5 in the dampening the host antibacterial response by targeting TBK1 in Hela cells (Zheng et al., 2016). And IpaH7.8 were reported to activates NLRP1B by degrading NLRP1B through its E3 ligase activity (Sandstrom et al., 2019). Here, the mechanisms of *Shigella*-induced caspase-1 activation and cell death were examined. We demonstrate that IpaH4.5 mediates NLRP3 protein stability, caspase-1 activation, IL-1β production, and cell death in *Shigella*-infected macrophages. Mutation of the IpaH4.5 E3 ligase motif not only abolishes NLRP3 inflammasome signaling but also dramatically reduces the ability of *Shigella* to induce cell pyroptosis. Our results identify a previously unrecognized role of the member of IpaH family in the regulation of inflammasome signaling and clarify the molecular basis for the cytosolic response to the T3SS effector.

## Results

### IpaH4.5 Protein Associates With NLRP3

**Figure 1:**
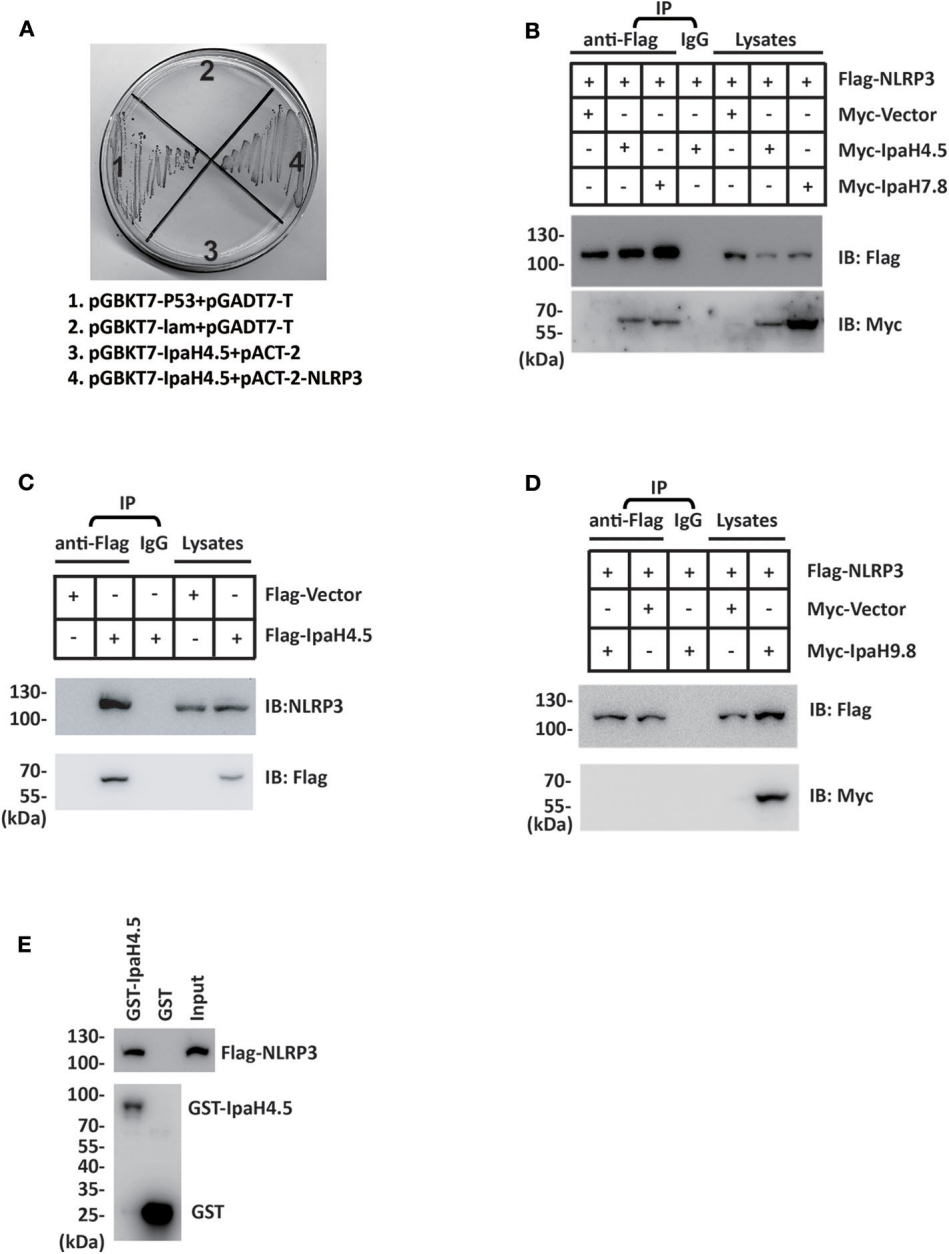
IpaH4.5 protein associates with NLRP3. **(A)** Yeast two-hybrid analysis in the AH109 yeast strain co-transformed with the indicated plasmids. IpaH4.5 was fused to GAL4-DNA binding domain (pGBKT7), and NRLP3 was fused with GAL4-transactivating domain (pACT2). A positive IpaH4.5-NRLP3 interaction resulted in colony formation on synthetic medium lacking tryptophan, leucine, adenine, and histidine containing X-gal. pGBKT7-TP53 + pGADT7-T control vector and pGBKT7-lam + pGADT7-T control vector were used as positive and negative controls, respectively. AH109 co-transfected with pGBKT7-IpaH4.5 + pACT-2 was used to exclude the self-activation of IpaH4.5. **(B)** Flag-tagged NLRP3 or the corresponding empty vector was transfected into HEK293 cells in the presence of Myc-tagged IpaH4.5 or Myc-tagged IpaH7.8. Then, coimmunoprecipitation experiments were performed, and anti-Flag or anti-Myc antibodies were used to probe anti-Flag or IgG immunoprecipitates. **(C)** Flag-tagged IpaH4.5 was transfected into RAW264.7 cells and coimmunoprecipitation experiments were performed. Anti-Flag or anti-NLRP3 antibodies were used to probe anti-Flag or IgG immunoprecipitates. **(D)** Flag-tagged NLRP3 or the corresponding empty vector was transfected into HEK293 cells in the presence of Myc-tagged IpaH9.8. Then, coimmunoprecipitation experiments were performed, and anti-Flag or anti-Myc antibodies were used to probe anti-Flag or IgG immunoprecipitates. **(E)** Flag-tagged NLRP3 was transfected into HEK293 cells. GST-tagged IpaH4.5 was subjected to a pull-down assay with the lysates of HEK293T cells. Immunoblotting analyses with anti-Flag antibody are shown at the top. Loading of the GST proteins assessed with anti-GST antibody are shown at the bottom. GST was used as a negative control. Images are representative of at least three independent experiments **(B–E)**, except for **(A)**, which were performed twice.

Our previous work showed that TANK-binding kinase 1 (TBK1) is a new target of the IpaH4.5 protein (Zheng et al., 2016). To explore the other possible host targets of IpaH4.5, a yeast two-hybrid (Y2H) assay was conducted, and a possible interaction between IpaH4.5 and NLRP3 was revealed (Figure 1A). Using HEK293 cells, we performed a coimmunoprecipitation experiment to confirm the results of the Y2H assay. The results showed that IpaH4.5 was detected in the anti-Flag immunoprecipitate from 293T cells cotransfected with Flag-NLRP3 but not with Flag-Vector (Figure 1B). The IpaH7.8 was used as a positive control (Suzuki et al., 2014). Consistently, the endogenous NLRP3 was detected in the anti-Flag immunoprecipitate from RAW264.7 cells transfected with Flag-Ipah4.5 but not with Flag-Vector (Figure 1C). Then, we explored the interaction of NLRP3 with the other IpaH family member, IpaH9.8. Coimmunoprecipitation experiments showed the no interaction between NLRP3 with IpaH9.8 (Figure 1D). Additionally, the interaction between IpaH4.5 and NLRP3 was further demonstrated by a GST pull-down assay (Figure 1E). These data suggest IpaH4.5 interact with NLRP3.

### IpaH4.5 Stimulates K63-Linked Ubiquitination of NLRP3

**Figure 2:**
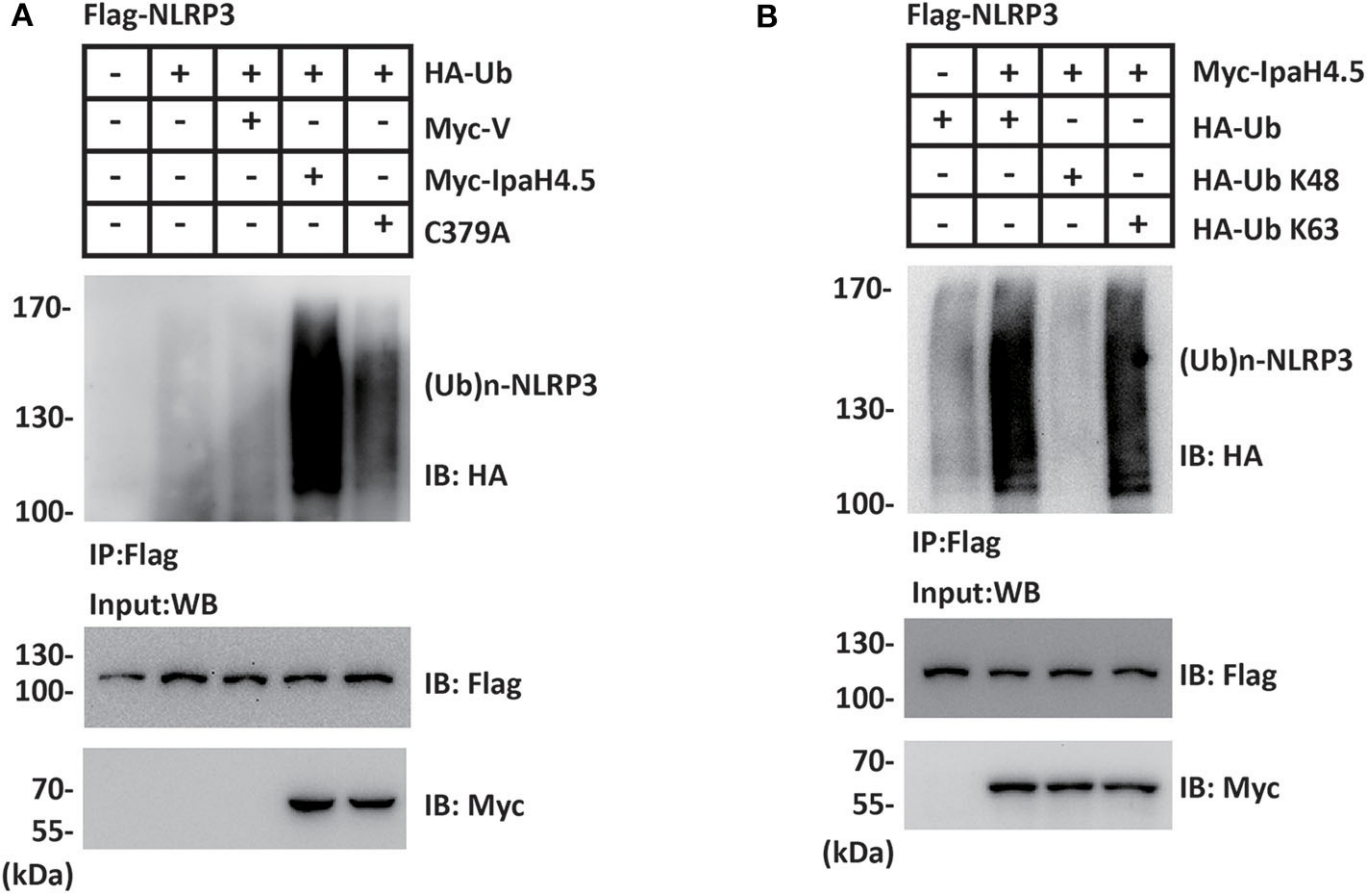
IpaH4.5 stimulates K63-linked ubiquitination of NLRP3. **(A)** Cells were transfected with Flag-NLRP3, HA-Ub together with Myc-vector, Myc-IpaH4.5, or Myc-IpaH4.5 C379A. NLRP3 protein was precipitated from cell lysates with an anti-Flag affinity gel, and the effect of IpaH4.5 or Myc-IpaH4.5 on NLRP3 ubiquitination was detected with an anti-HA antibody. For input, cell lysates were blotted with anti-Flag and anti-Myc antibodies. **(B)** Cells expressing Flag-NLRP3 and Myc-IpaH4.5 in the presence of HA-tagged ubiquitin, HA-tagged K48 ubiquitin (Ub K48) or HA-tagged K63 ubiquitin (Ub K63) were precipitated with anti-Flag antibody and blotted with anti-HA and anti-Flag antibodies. For input, cell lysates were blotted with anti-Myc antibody. Images are representative of at least three independent experiments.

To investigate whether NLRP3 is a substrate of IpaH4.5 E3 ubiquitin ligase, HEK293 cells were cotransfected Flag-NLRP3, HA-Ub together with Myc-vector, Myc-IpaH4.5, or Myc-IpaH4.5 C379A. NLRP3 protein was precipitated from cell lysates with an anti-Flag affinity gel, and the effect of IpaH4.5 or Myc-IpaH4.5 on NLRP3 ubiquitination was detected with an anti-HA antibody. The results showed that IpaH4.5 led to a sharp increase in the level of NLRP3 ubiquitination (Figure 2A). Specifically, mutation of the IpaH4.5 E3 ligase motif (C379A mutant) did not upregulate NLRP3 ubiquitination to the same intensity as wildtype IpaH4.5 (Figure 2A), indicating the importance of Cys 379 active site in regulating NLRP3 ubiquitination. The two most adequately characterized forms of polyubiquitination occur at lysine 48 (K48) and lysine 63 (K63). Therefore, we transfected HA-Ub K48 or HA-Ub K63 into HEK293 cells in the presence of Flag-NLRP3 and Myc-IpaH4.5. The coimmunoprecipitation assay results showed that the ubiquitination of NLRP3 was catalyzed by IpaH4.5 mainly at position K63 (Figure 2B).

### IpaH4.5 Mediates the Stabilization of NLRP3

**Figure 3:**
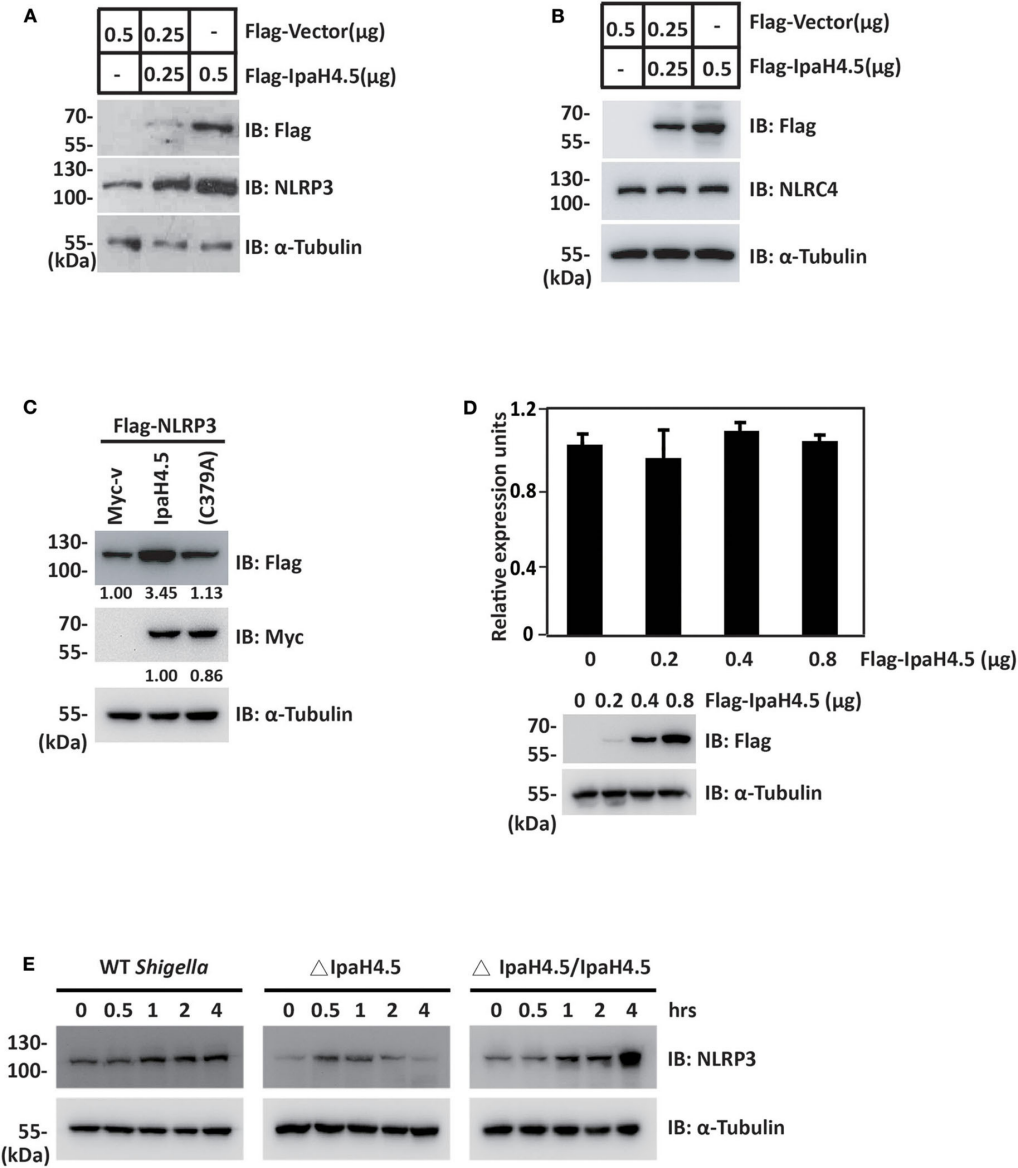
IpaH4.5 mediates the stabilization of NLRP3. **(A)** Immunoblot analysis of NLRP3 protein expression in RAW264.7 cells transfected with indicated doses of Flag-Vector and Flag-IpaH4.5 plasmid. α-Tubulin was utilized to ensure equal amounts of protein loading. **(B)** Immunoblot analysis of NLRC4 protein expression in RAW264.7 cells transfected with indicated doses of Flag-Vector and Flag-IpaH4.5 plasmid. α-Tubulin was utilized to ensure equal amounts of protein loading. **(C)** Immunoblot analysis of NLRP3 protein expression in HEK293 cells transfected with Flag-NLRP3 in the presence of Myc-IpaH4.5 or Myc-IpaH4.5 C379A. α-Tubulin was utilized to ensure equal amounts of protein loading. **(D)** Comparison of NLRP3 mRNA levels in RAW264.7 cells 24 h post-transfected with different doses of IpaH4.5 plasmid (0, 0.2, 0.4, and 0.8 μg). The expression levels are fold change relative to the 0 μg treatment. The experiments were performed independently at least three times with comparable results and the error bars are presented as the means ± SEMs. **(E)** BMDMs were infected with different background of *Shigella* or ΔIpaH4.5 mutant *Shigella* strains, NLRP3 protein expression was detected by immunoblot analysis at the indicated time points. α-Tubulin was utilized to ensure equal amounts of protein loading. Gel images are representative of at least three independent experiments. Data in **(D)** is representative of at least three independent experiments. Data sets were analyzed using Student's *t*-test.

Since IpaH4.5 mediates the activation of K63-linked ubiquitination of NLRP3, we next determined the effect of IpaH4.5 on NLRP3 expression. To this end, increasing amounts of plasmid encoding IpaH4.5 were transfected into RAW264.7 cells. The results showed that overexpression of IpaH4.5 led to a substantial increase in the concentration of endogenous NLRP3 protein levels (Figure 3A). In contrast, IpaH4.5 did not affect the level of the transfected ICE protease-activating factor NLRC4 protein (Figure 3B). Specifically, IpaH4.5 E3 ligase activity was required, as the IpaH4.5 C379A mutant had compromised activity in inducing NLRP3 upregulation (Figure 3C). Quantitative RT-PCR showed that NLRP3 mRNA levels were not affected by IpaH4.5 overexpression (Figure 3D), suggesting that IpaH4.5 stabilized NLRP3 protein by posttranscriptional modification. We then assessed whether IpaH4.5 is able to mediate the stabilization of endogenous NLRP3 under physiological conditions by analysis of NLRP3 protein kinetics in response to bacterial infection, and observed that NLRP3 protein levels increased in WT *Shigella*–infected BMDMs (Figure 3E). Furthermore, the endogenous level of NLRP3 in cells infected with ΔIpaH4.5 bacteria was significantly lower than that of NLRP3 in cells infected with WT and complemented bacteria (Figure 3E). Taken together, these data indicate that IpaH4.5 mediates the stabilization of NLRP3 during *Shigella* infection.

### IpaH4.5 Stimulates NLRP3-Mediated Inflammasome Activation

**Figure 4:**
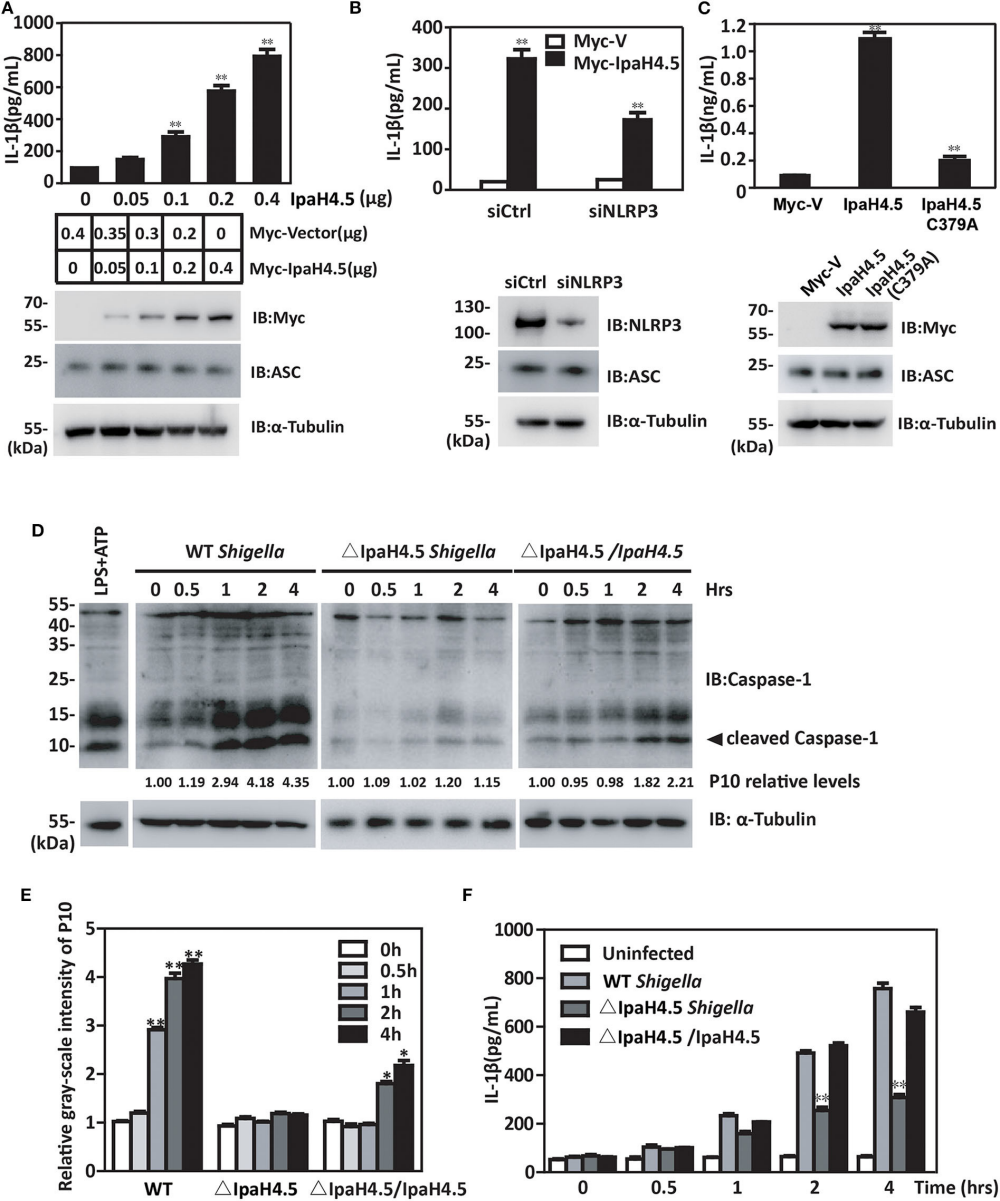
IpaH4.5 stimulating NLRP3-mediated inflammasome activation. **(A)** ELISA analysis of IL-1β in the culture medium of RAW264.7 cells cotransfected with Flag-ASC and indicated doses of IpaH4.5. **(B)** ELISA analysis of IL-1β in supernatant of WT and siNLRP3 RAW264.7 cells cotransfected with expression plasmids encoding IpaH4.5 and ASC. **(C)** ELISA analysis of IL-1β in the culture medium of RAW264.7 cells cotransfected with expression plasmids encoding ASC, IpaH4.5, or IpaH4.5 C379A. **(D)** Immunoblot showing cleavage of procaspase-1 in BMDM cells infected with wild-type or ΔIpaH4.5 mutant *Shigella* strains for the indicated times. The LPS was used as a positive control to induce the cleavage of procaspase-1. **(E)** The relative gray-scale intensity of P10. The densitometry of P10 is relative to each full-length caspase-1. The experiments were performed independently at least three times with comparable results and the error bars are presented as the means ± SEMs. **(F)** ELISA analysis of IL-1β in the culture medium of BMDM cells infected with the indicated *Shigella* strains for the indicated times. Gel images are representative of at least three independent experiments **(D)**. Data in **(A–C,E,F)** is representative of at least three independent experiments. The data are presented as the means ± SEMs. Student's *t*-test was used for statistical analysis: ^*^P < 0.05; ^**^P < 0.01.

The finding that IpaH4.5 mediates the stabilization of NLRP3 prompted us to investigate whether IpaH4.5 is involved in NLRP3-mediated inflammasome activation. RAW264.7 cells are naturally ASC-negative (Pelegrin et al., 2008), so they need to be transfected with ASC to get inflammasome activation and IL-1β secretion. IpaH4.5 overexpression in RAW264.7 cells which were cotransfected with Flag-ASC significantly increased IL-1β secretion in a dose-dependent manner (Figure 4A). Specifically, inhibition of NLRP3 by siRNA in RAW264.7 cells overexpressed ASC significantly abolished the enhancement of IpaH4.5-induced IL-1β secretion (Figure 4B). Furthermore, IL-1β production was specifically upregulated by WT IpaH4.5 but not the C379A mutant (Figure 4C), demonstrating the importance of IpaH4.5 E3 ligase activity in inflammasome activation.

We subsequently examined whether IpaH4.5 could mediate the activation of the NLRP3 inflammasome under physiological conditions. We observed significant caspase-1 activation consistent with the relative gray-scale show (Figures 4D,E) and IL-1β production (Figure 4F) in WT *Shigella*-infected BMDM cells. Interestingly, bacteria lacking IpaH4.5 (ΔIpaH4.5) induced significantly reduced caspase-1 activation (Figure 4D) and IL-1β production (Figure 4F). Taken together, these data indicate that *Shigella*-induced inflammasome activation requires the E3 ligase activity of IpaH4.5.

### IpaH4.5 Contributes to Pyroptosis in Macrophages Infected With *Shigella*

**Figure 5:**
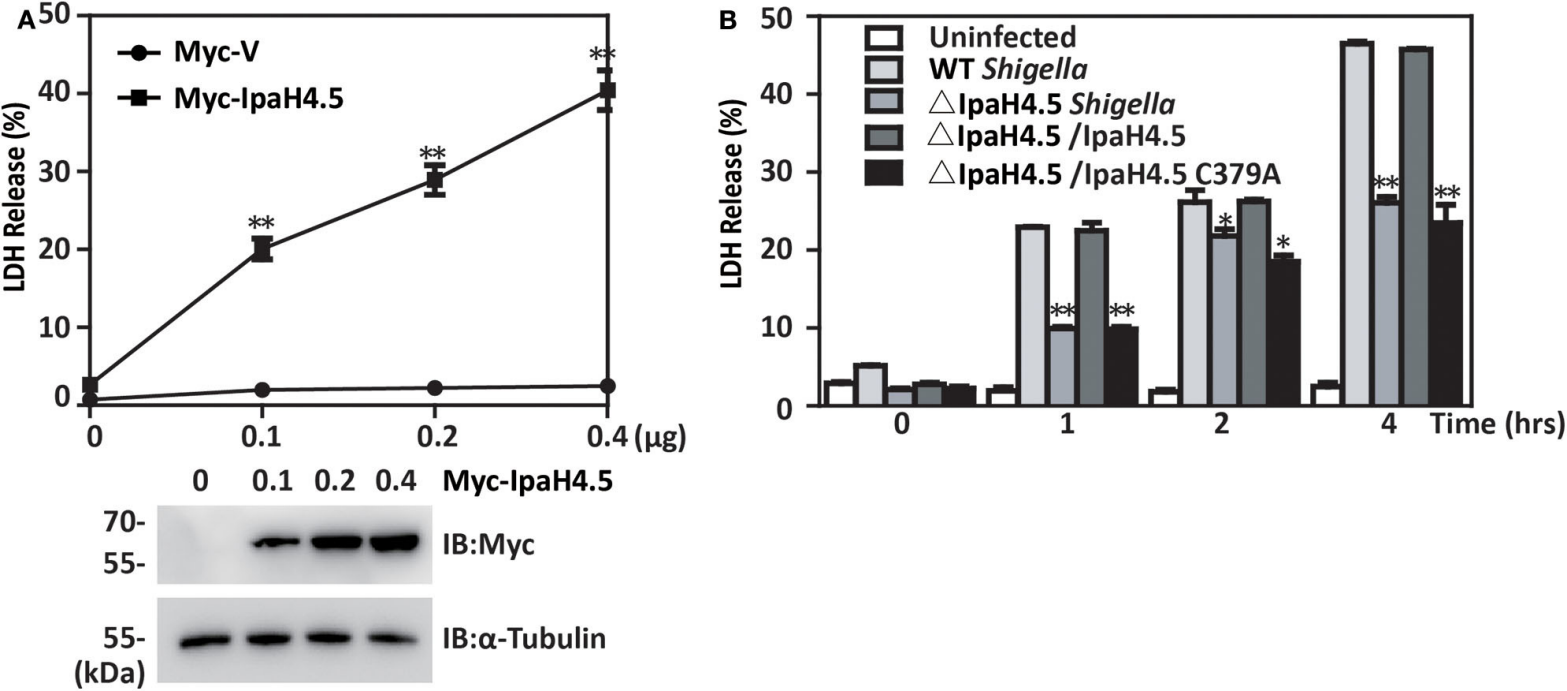
IpaH4.5 contributes to pyroptosis in macrophages infected with *Shigella*. **(A)** LDH was measured in the culture medium of RAW264.7 cells 48 h posttransfected with increasing doses of IpaH4.5 and ASC. **(B)** LDH was measured in the culture medium of BMDM cells infected with the indicated *Shigella* strains for the indicated times. Images are representative of at least three independent experiments. The data are presented as the means ± SEMs. Student's *t*-test was used for statistical analysis: ^*^P < 0.05; ^**^P < 0.01.

We next investigated the role of IpaH4.5 in *Shigella*-induced cell death. We found that overexpression of IpaH4.5 in RAW264.7 macrophages significantly increased the release of lactate dehydrogenase (LDH), which is a marker of pyroptosis (Figure 5A). In addition, BMDMs were infected with wild-type *Shigella* and the IpaH4.5 mutant (ΔIpaH4.5), and cell death was determined by LDH release in infected cells. We observed significantly lower LDH release in BMDMs infected with IpaH4.5-deficient *Shigella* when compared to that of WT and ΔIpaH4.5/IpaH4.5 *Shigella* (Figure 5B). Furthermore, ΔIpaH4.5/IpaH4.5 (C379A) *Shigella* infected BMDMs was failed to increase LDH release. Consistently, the flow cytometry analysis showed ΔIpaH4.5 mutant caused less pyroptosis than wild-type *Shigella* and ΔIpaH4.5/IpaH4.5 *Shigella* (Supplementary Figure 1). Taken together, these data suggesting that IpaH4.5 E3 ligase activity is important in *Shigella*-induced cell death.

## Discussion

We have identified a previously unrecognized effect of the *Shigella* Type III secretion effector IpaH4.5 in the regulation of inflammasome signaling (Zheng et al., 2016). Previous work had established the molecular function of IpaH7.8 in the context of activating inflammasomes by targeting glomulin as an E3 ubiquitin ligase (Suzuki et al., 2014). However, the other potential host targets of IpaH family members have remained unclear. In this study, we reveal that NLRP3 is a novel target of IpaH4.5. Our results clearly demonstrate the importance of IpaH4.5 in the process of caspase-1 activation and pyroptosis. There is some evidence to verify this conclusion. First, IpaH4.5 was proven to interact directly with NLRP3. Second, IpaH4.5 upregulated NLRP3 protein stability by increasing the K63-linked ubiquitination level of NLRP3, and Cys 379 was the key active site of IpaH4.5 in regulating NLRP3 ubiquitination. Third, IpaH4.5-deficient bacteria displayed reduced NLRP3 inflammasome activation and cell death. In summary, these data suggest that the cellular expression level of NLRP3 that is regulated by IpaH4.5 E3 ligase activity is important for *Shigella*-mediated inflammasome activation and cell death.

*Shigella* stimulates IL-1β secretion by delivering various effectors into host cells via the T3SS. IpaB, a T3SS effector of *Shigella*, assembles an ion channel within cell membrane to allow for potassium influx, which is recognized by the NLRC4-inflammasome and triggers IL-1β secretion (Senerovic et al., 2012). A recent study showed *Shigella* induces rapid macrophage pyroptosis by delivering the IpaH7.8 activating NLRP3 and NLRC4 inflammasomes and IL-1β secretion (Suzuki et al., 2014). NLRs can be partitioned into three subfamilies according to their phylogenetic relationships, including NOD, NLRP and NLRC4 subfamilies. Multiple NLRs are involved in S. *flexneri* infection (Cervantes et al., 2008). Both the NLRC4 inflammasome and NLRP3 inflammasome were reported to mediate the process of caspase-1 activation, pyroptosis, and even necrosis (Mariathasan et al., 2004). However, the specific factors detected as PAMPs and the direct interaction between NLRP3 and PAMPs have not yet been identified. We have known that the expression of NLRP3 and the assembly of the NLRP3 inflammasome are two indispensable processes in the activation and regulation of the NLRP3 inflammasome (Yang et al., 2015). Here, we found that IpaH4.5, an inherent *Shigella* product, is able to upregulate NLRP3 expression and activate the NLRP3 inflammasome. Therefore, as a NLRP3 agonist, IpaH4.5 itself is adequate for NLRP3 inflammasome activation.

Further resolved is the involvement of the T3SS effector IpaH4.5 in *Shigella*-induced cell death. IpaB, a virulence factor secreted by *Shigella* T3SS, was reported to induce cell death by directly binding to caspase-1 (Espina et al., 2006). Additionally, IpaB is important for effector proteins to be injected into host cells *via* the T3SS, owing to its indispensable role in the assembly of the T3SS transmembrane pore complex (Suzuki et al., 2007). Recent reports suggest that IpaH7.8-mediated glomulin degradation during *Shigella* infection activated NLR inflammasomes and promoted cell death (Suzuki et al., 2014). Here, we showed that IpaH4.5 contributes to both inflammasome activation and pyroptosis in macrophages infected with *Shigella*. The data presented here directly link the bacterial T3SS effector with the initiation of inflammation through NLRP3 and illuminate the molecular mechanism for the cellular response to the T3SS effector. Ipah4.5-NRLP3 interaction may be a potential therapeutic target for inflammasome-related illnesses.

## Materials and Methods

### Yeast Two-Hybrid Screening

To perform the yeast two-hybrid screening, IpaH4.5 was inserted into the bait pGBKT7 vector for expression as a fusion protein with the Gal4 DNA binding domain (Gal4-BD). This plasmid was used to transform the AH109 yeast strain, and Gal4-BD fused IpaH4.5 was used as bait for the screening of human spleen cDNA library. The spleen cDNA library was inserted into the pGADT7 vector (Clontech) for expression as fusions with the Gal4 activation domain (Gal4-AD) and was maintained in the Y187 strain of yeast. Transformed AH109 and Y187 yeast cells were mixed together for mating. Positive clones were selected on synthetic dropout medium lacking 4 nutrients (Leu/Trp/Ade/His). The blue colonies were kept, and the positive results were confirmed by repeating assays. cDNA plasmids isolated from positive colonies were introduced into Escherichia coli DH5α and sequenced. The sequences were analyzed with the BLAST program in NCBI.

### Strains and Plasmids

The *Shigella flexneri* strain 2a 301 was utilized as the wild-type (WT). The ΔIpaH4.5 and ΔIpaH4.5/IpaH4.5, ΔIpaH4.5/IpaH4.5 C379A strain were constructed as described previously (Zheng et al., 2016). In brief, the *Shigella* ipaH4.5 gene was removed by allelic exchange using a modification of the γ-red recombinase-mediated recombination system. IpaH4.5 or IpaH4.5 C379A (residue Cys379 was replaced by Ala) was cloned into pACYC184 to generate pACYC184-IpaH4.5 or pACYC184-IpaH4.5 C379A. The resultant plasmids were introduced into *Shigella* ΔIpaH4.5 strain. The IpaH4.5 or IpaH4.5 C379A coding sequence was amplified by PCR and cloned into Ampicillin-resistance vector pcDNA3-Flag, pCMV-Myc, and pGEX4T-1-GST. GST fusion proteins were generated by expression in pGEX4T-1-based vectors (Amersham Biosciences Biotech) in *E.coli* BL21 (DE3). Other plasmids were preserved in our lab at −20°C.

### Cell Culture and Transfections

HEK293 and RAW264.7 cells were cultured in Dulbecco's modified Eagle's medium (Gibco) supplemented with 10% fetal bovine serum (HyClone), 100 units/mL penicillin, 100 μg/mL streptomycin and 2 mM L-glutamine. Transient transfections were performed with jetPRIME reagent (Polyplus) or Trans IT-X2 (Mirus) following the manufacturer's instructions. The siRNA nucleotide sequences targeting NLRP3 were as follows: 5' -CTAGCAGATCTCATTATCA -3'.

### Bone Marrow-Derived Macrophage (BMDM) Isolation

Bone marrow cells from femur exudates of C57BL/6 were grown at 37°C in a humidified incubator in RPMI 1640 medium (RPMI, Invitrogen) containing 10% FBS, 2 mM L-glutamine, 100 U/ml penicillin, 100 mg/ml streptomycin, and 20 ng/ml mouse M-CSF (Peprotech, Rocky Hill, NJ, USA) for 7 days. Differentiated BMDMs were replated into six-well dishes 18 h before infection.

### Bacterial Infection

The *Shigella* were cultured in MCA medium containing 0.01% Congo red and the single bacteria colony was cultured in LB fluid medium. Infection of BMDM cells with different strains of *Shigella* was carried out as described previously. In brief, the cells were infected with *Shigella* at an MOI of 10. Strains and cells were centrifuged together at a speed of 700 × g for 10 min at room temperature. After incubated 30 min at 37°C in 5%, the infected cells were washed with PBS for three times and 100 μg/mL gentamicin and 60 μg/mL kanamycin were used in fresh medium to kill extracellular bacteria. At the indicated times postinfection, the LDH activity and the IL-1βin the culture supernatants of infected cells was measured according to the manufacturer's protocol and the cell lysates were subjected to western blotting to verify the success of bacterial infection.

### Immunoprecipitation

Cell lysates were incubated 2 h with anti-Flag M2 affinity gel (Sigma) or anti-mouse IgG-conjugated beads (Sigma) at 4°C. An aliquot of cell lysates (10%) without antibody added was used as a control for immunoprecipitation. Then, the beads were collected and washed three times with lysis buffer (50 mM Tris-HCl at pH 7.5, 1 mM EDTA, 0.3 mM dithiothreitol, 150 mM NaCl, 0.1% NP40), followed by adding SDS-PAGE loading buffer for the preparation of immunoblot samples.

### Immunoblot Analysis

Protein or immunoprecipitation samples were subjected to SDS-PAGE and transferred onto the PVDF membrane (Millipore). Then, primary antibodies were incubated with the PVDF membrane at a dilution of 1:1000, and HRP-conjugated secondary antibodies at a dilution of 1:5000 and ECL reagent (PerkinElmer Life Sciences) were used for the detection of antigen-antibody complexes. The primary antibodies are as follows: anti-NLRP3 (Antipogen, AG-20B-0014-C100), anti-NLRC4 (ABclonal, A7382), anti-caspase-1 (SANTA, sc-515), anti-Myc (Proteintech, 60003-2-Ig), anti-Flag (Sigma, A8549), anti-HA (sigma, H9658), anti-GST (Proteintech, HRP-66001) and anti-α-Tubulin (Sigma, T6074) antibodies.

### GST Pull-Down Assay

Purified GST-IpaH4.5 fusion protein or GST was bound to glutathione beads to capture Flag-NLRP3 in the cell lysates. After three washes, the adsorbates were subjected to SDS-PAGE, and immunoblot analysis was performed with an anti-Flag antibody to detect the interaction of Flag-NLRP3 and the GST-IpaH4.5 fusion protein.

### *In vivo* Ubiquitination Assays

The vivo ubiquitination assay was performed as described previously (Wang et al., 2013; Wei et al., 2016). In brief, HEK293 cells were transfected with Myc-vector, Myc-IpaH4.5, or IpaH4.5 C379A, Flag-NLRP3, and HA-tagged ubiquitin (HA-Ub), HA-Ub K48 or HA-Ub K63 plasmid in combination. At 48 h post-transfection, the cells were treated with MG132 (20 mM, 6 h) and harvested, lysed using ultrasonication after added buffer (50 mM Tris-HCl at pH 7.5, 1 mM EDTA, 0.3 mM dithiothreitol, 150 mM NaCl, 0.1% NP40, 1 mM PMSF). The cell lysates were collected into two aliquots. One aliquot was used for conventional immunoblotting. The other aliquot were used for the immunoprecipitation with anti-Flag antibody and analyzed by immunoblotting.

### Quantitative RT-PCR

RAW264.7 cells were lysed in Trizol (Invitrogen) and mRNA was extracted. The cDNA was generated from total RNA using random priming and Moloney murine leukemia virus reverse transcriptase (Invitrogen). The cDNA was used for qPCR using primers specific for NLRP3. The values obtained by qPCR were normalized based on β-actin. NRLP3 forward primer:5'- GATCTTCGCTGCGATCAACAG-3'; NRLP3 reverse primer: 5'- CGTGCATTATCTGAACCCCAC-3'.

### Cytotoxicity Assay

An LDH release assay was performed according to the manufacturer's instructions (LDH-Cytox Assay Kit, BioLegend). The absorbance was measured at 490 nm, and values are expressed as percentages of the 100% lysis control. All values are normalized to the uninfected control.

### ELISA (Enzyme-Linked Immunosorbent Assay)

The treated cell supernatant was collected by centrifuging at 3,000 r/min for 30 min to remove cellular debris. IL-1β concentration was assayed by ELISA Kit (DKW 1210122; Dakewe, Shenzhen, Guangzhou, China) according to the manufacturer's instructions.

### Flow Cytometry Analysis

The BMDM cells were harvested at indicated time postinfection, washed with PBS twice and stained with PI and Annexin V following the manufacturer's introductions. After incubation at room temperature for 15 min in the dark, the stained cells were analyzed on the flow cytometer (Invitrogen).

### Statistical Analysis

Data are presented as the mean ± SE. The differences between groups were analyzed by two-tailed Student's *t*-test.

## Data Availability Statement

The datasets generated for this study are available on request to the corresponding author.

## Ethics Statement

The animal study was reviewed and approved by the Institutional Animal Care Committee of Beijing Institute of Biotechnology.

## Author Contributions

HZ, BW, MD, and CW conceived, designed the study, edited, and revised the manuscript. JS, XW, HL, LW, BW, MD, and CW conducted the experiments and wrote the first draft of the manuscript. XiaopanY, MM, XiaoliY, and KG analyzed the data. ZS, XH, BW, MD, and YZ helped in statistical analyses. All authors read and approved the final manuscript.

## Conflict of Interest

The authors declare that the research was conducted in the absence of any commercial or financial relationships that could be construed as a potential conflict of interest.

## Supplementary Material

Supplementary Figure 1 Flow cytometry analysis of macrophages pyroptosis induced by IpaH4.5. BMDM cells were collected after infected 2 h and analyzed by flow cytometry. Images are representative of at least three independent experiments.

Supplementary Table 1 Strains and plasmids used for this study.

## References

[B1] Ashida H. Kim M. Schmidt-Supprian M. Ma A. Ogawa M. Sasakawa C. (2010). A bacterial E3 ubiquitin ligase IpaH9.8 targets NEMO/IKKgamma to dampen the host NF-kappaB-mediated inflammatory response. Nat. Cell Biol. 12, 66–73. 10.1038/ncb2006 20010814 PMC3107189

[B2] Ashida H. Sasakawa C. (2015). Shigella IpaH family effectors as a versatile model for studying pathogenic bacteria. Front. Cell Infect. Microbiol. 5:100. 10.3389/fcimb.2015.00100 26779450 PMC4701945

[B3] Buttner C. R. Sorg I. Cornelis G. R. Heinz D. W. Niemann H. H. (2008). Structure of the *Yersinia enterocolitica* type III secretion translocator chaperone SycD. J. Mol. Biol. 375, 997–1012. 10.1016/j.jmb.2007.11.009 18054956

[B4] Caruso R. Warner N. Inohara N. Nunez G. (2014). NOD1 and NOD2: signaling, host defense, and inflammatory disease. Immunity 41, 898–908. 10.1016/j.immuni.2014.12.010 25526305 PMC4272446

[B5] Cervantes J. Nagata T. Uchijima M. Shibata K. Koide Y. (2008). Intracytosolic *Listeria monocytogenes* induces cell death through caspase-1 activation in murine macrophages. Cell Microbiol. 10, 41–52. 10.1111/j.1462-5822.2007.01012.x 17662073

[B6] Dadi H. Jones T. A. Merico D. Sharfe N. Ovadia A. Schejter Y.. (2018). Combined immunodeficiency and atopy caused by a dominant negative mutation in caspase activation and recruitment domain family member 11 (CARD11). J. Allergy Clin. Immunol. 141, 1818–1830. e1812. 10.1016/j.jaci.2017.06.047 28826773

[B7] Espina M. Olive A. J. Kenjale R. Moore D. S. Ausar S. F. Kaminski R. W.. (2006). IpaD localizes to the tip of the type III secretion system needle of *Shigella flexneri*. Infect. Immun. 74, 4391–4400. 10.1128/IAI.00440-06 16861624 PMC1539624

[B8] Franchi L. Park J. H. Shaw M. H. Marina-Garcia N. Chen G. Kim Y. G.. (2008). Intracellular NOD-like receptors in innate immunity, infection and disease. Cell Microbiol. 10, 1–8. 10.1111/j.1462-5822.2007.01059.x 17944960

[B9] Fukata M. Vamadevan A. S. Abreu M. T. (2009). Toll-like receptors (TLRs) and Nod-like receptors (NLRs) in inflammatory disorders. Semin. Immunol. 21, 242–253. 10.1016/j.smim.2009.06.005 19748439

[B10] Hamada M. Shoguchi E. Shinzato C. Kawashima T. Miller D. J. Satoh N. (2013). The complex NOD-like receptor repertoire of the coral *Acropora digitifera* includes novel domain combinations. Mol. Biol. Evol. 30, 167–176. 10.1093/molbev/mss213 22936719

[B11] Jamilloux Y. Pierini R. Querenet M. Juruj C. Fauchais A. L. Jauberteau M. O.. (2013). Inflammasome activation restricts *Legionella pneumophila* replication in primary microglial cells through flagellin detection. Glia 61, 539–549. 10.1002/glia.22454 23355222

[B12] Kanneganti T. D. Lamkanfi M. Nunez G. (2007). Intracellular NOD-like receptors in host defense and disease. Immunity 27, 549–559. 10.1016/j.immuni.2007.10.002 17967410

[B13] Maharana J. Vats A. Gautam S. Nayak B. P. Kumar S. Sendha J.. (2017). POP1 might be recruiting its type-Ia interface for NLRP3-mediated PYD-PYD interaction: insights from MD simulation. J. Mol. Recognit. 30:2632. 10.1002/jmr.2632 28370480

[B14] Mariathasan S. Newton K. Monack D. M. Vucic D. French D. M. Lee W. P.. (2004). Differential activation of the inflammasome by caspase-1 adaptors ASC and Ipaf. Nature 430, 213–218. 10.1038/nature02664 15190255

[B15] Menard R. Sansonetti P. J. Parsot C. (1993). Nonpolar mutagenesis of the ipa genes defines IpaB, IpaC, and IpaD as effectors of *Shigella flexneri* entry into epithelial cells. J. Bacteriol. 175, 5899–5906. 10.1128/JB.175.18.5899-5906.1993 8376337 PMC206670

[B16] Otsubo R. Mimuro H. (2019). Shigella effector IpaH4.5 targets 19S regulatory particle subunit RPN13 in the 26S proteasome to dampen cytotoxic T lymphocyte activation. Cell Microbiol. 21:e12974. 10.1111/cmi.12974 30414351

[B17] Pelegrin P. Barroso-Gutierrez C. Surprenant A. (2008). P2X7 receptor differentially couples to distinct release pathways for IL-1beta in mouse macrophage. J. Immunol. 180, 7147–7157. 10.4049/jimmunol.180.11.7147 18490713

[B18] Prajapati B. Jena P. K. Rajput P. Purandhar K. Seshadri S. (2014). Understanding and modulating the Toll like Receptors (TLRs) and NOD like Receptors (NLRs) cross talk in type 2 diabetes. Curr. Diabetes Rev. 10, 190–200. 10.2174/1573399810666140515112609 24828062

[B19] Rohde J. R. Breitkreutz A. Chenal A. Sansonetti P. J. Parsot C. (2007). Type III secretion effectors of the IpaH family are E3 ubiquitin ligases. Cell Host Microbe. 1, 77–83. 10.1016/j.chom.2007.02.002 18005683

[B20] Sandstrom A. Mitchell P. S. Goers L. Mu E. W. (2019). Functional degradation: a mechanism of NLRP1 inflammasome activation by diverse pathogen enzymes. Science 364:6435. 10.1126/science.aau1330 30872533 PMC6532986

[B21] Senerovic L. Tsunoda S. P. Goosmann C. Brinkmann V. Zychlinsky A. Meissner F.. (2012). Spontaneous formation of IpaB ion channels in host cell membranes reveals how Shigella induces pyroptosis in macrophages. Cell Death Dis. 3:e384. 10.1038/cddis.2012.124 22951981 PMC3461361

[B22] Srinivasula S. M. Poyet J. L. Razmara M. Datta P. Zhang Z. Alnemri E. S. (2002). The PYRIN-CARD protein ASC is an activating adaptor for caspase-1. J. Biol. Chem. 277, 21119–21122. 10.1074/jbc.C200179200 11967258

[B23] Suzuki S. Mimuro H. Kim M. Ogawa M. Ashida H. Toyotome T.. (2014). Shigella IpaH7.8 E3 ubiquitin ligase targets glomulin and activates inflammasomes to demolish macrophages. Proc. Natl. Acad. Sci. U. S. A. 111, E4254–E4263. 10.1073/pnas.1324021111 25246571 PMC4210038

[B24] Suzuki T. Franchi L. Toma C. Ashida H. Ogawa M. Yoshikawa Y.. (2007). Differential regulation of caspase-1 activation, pyroptosis, and autophagy via Ipaf and ASC in Shigella-infected macrophages. PLoS Pathog. 3:e111. 10.1371/journal.ppat.0030111 17696608 PMC1941748

[B25] Taghavi M. Khosravi A. Mortaz E. Nikaein D. Athari S. S. (2017). Role of pathogen-associated molecular patterns (PAMPS) in immune responses to fungal infections. Eur. J. Pharmacol. 808, 8–13. 10.1016/j.ejphar.2016.11.013 27851904

[B26] Takagi K. Kim M. Sasakawa C. Mizushima T. (2016). Crystal structure of the substrate-recognition domain of the Shigella E3 ligase IpaH9.8. Acta Crystallogr. F Struct. Biol. Commun. 72, 269–275. 10.1107/S2053230X16002715 27050259 PMC4822982

[B27] Thompson M. R. Kaminski J. J. Kurt-Jones E. A. Fitzgerald K. A. (2011). Pattern recognition receptors and the innate immune response to viral infection. Viruses 3, 920–940. 10.3390/v3060920 21994762 PMC3186011

[B28] Vourc'h P. Moreau T. Arbion F. Marouillat-Vedrine S. Muh J. P. Andres C. (2003). Oligodendrocyte myelin glycoprotein growth inhibition function requires its conserved leucine-rich repeat domain, not its glycosylphosphatidyl-inositol anchor. J. Neurochem. 85, 889–897. 10.1046/j.1471-4159.2003.01764.x 12716421

[B29] Wang F. Jiang Z. Li Y. He X. Zhao J. Yang X.. (2013). Shigella flexneri T3SS effector IpaH4.5 modulates the host inflammatory response via interaction with NF-κB p65 protein. Cell Microbiol. 15, 474–485. 10.1111/cmi.12052 23083102

[B30] Watarai M. Funato S. Sasakawa C. (1996). Interaction of Ipa proteins of *Shigella flexneri* with α5β1 integrin promotes entry of the bacteria into mammalian cells. J. Exp. Med. 183, 991–999. 10.1084/jem.183.3.991 8642302 PMC2192368

[B31] Wei C. Wang Y. Du Z. Guan K. Cao Y. Yang H.. (2016). The Yersinia Type III secretion effector YopM Is an E3 ubiquitin ligase that induced necrotic cell death by targeting NLRP3. Cell Death Dis. 7:e2519. 10.1038/cddis.2016.413 27929533 PMC5260993

[B32] Yang C. S. Kim J. J. Kim T. S. Lee P. Y. Kim S. Y. Lee H. M.. (2015). Small heterodimer partner interacts with NLRP3 and negatively regulates activation of the NLRP3 inflammasome. Nat. Commun. 6:6115. 10.1038/ncomms7115 25655831 PMC4347017

[B33] Yazdi A. S. Guarda G. Riteau N. Drexler S. K. Tardivel A. Couillin I.. (2010). Nanoparticles activate the NLR pyrin domain containing 3 (Nlrp3) inflammasome and cause pulmonary inflammation through release of IL-1α and IL-1β. Proc. Natl. Acad. Sci. U. S. A. 107, 19449–19454. 10.1073/pnas.1008155107 20974980 PMC2984140

[B34] Ye Z. Lich J. D. Moore C. B. Duncan J. A. Williams K. L. Ting J. P. (2008). ATP binding by monarch-1/NLRP12 is critical for its inhibitory function. Mol. Cell Biol. 28, 1841–1850. 10.1128/MCB.01468-07 18160710 PMC2258772

[B35] Zheng Z. Wei C. Guan K. Yuan Y. Zhang Y. Ma S.. (2016). Bacterial E3 ubiquitin ligase IpaH4.5 of *Shigella flexneri* targets TBK1 To dampen the host antibacterial response. J. Immunol. 196, 1199–1208. 10.4049/jimmunol.1501045 26700764

[B36] Zuliani-Alvarez L. Piccinini A. M. Midwood K. S. (2017). Screening for novel endogenous inflammatory stimuli using the secreted embryonic alkaline phosphatase NF-κB reporter assay. Bio. Protoc. 7:2220. 10.21769/BioProtoc.2220 28405594 PMC5386139

