# Supplementary figures and images for: The Shigella Type III Secretion Effector IpaH4.5 Targets NLRP3 to Activate Inflammasome Signaling
Source: Front Cell Infect Microbiol. 2020 Sep 30;10:511798. doi: 10.3389/fcimb.2020.511798 (PMC7561375; doi:10.3389/fcimb.2020.511798)

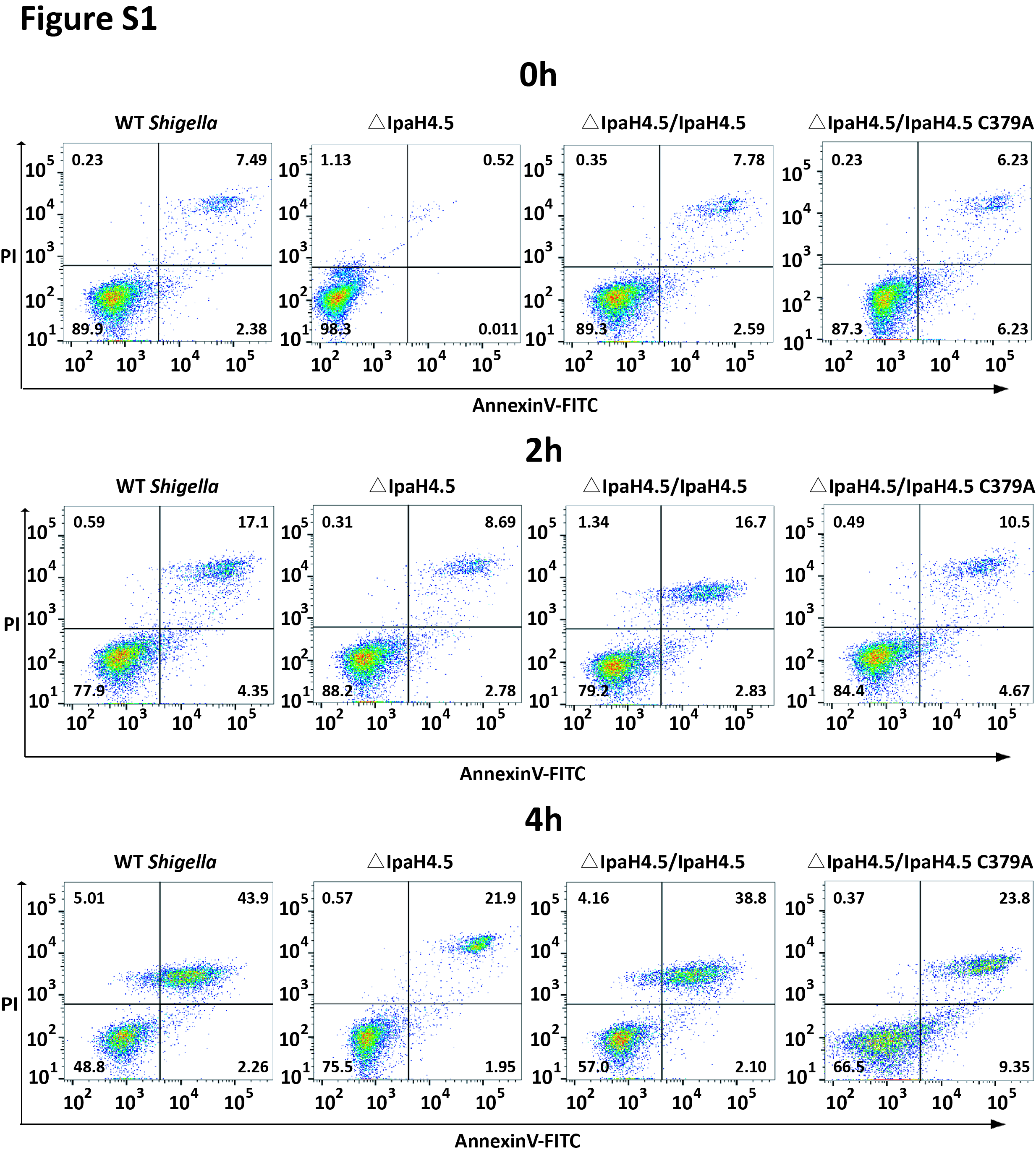

Supplement: Supplementary Figure 1 — Flow cytometry analysis of macrophages pyroptosis induced by IpaH4.5. BMDM cells were collected after infected 2 h and analyzed by flow cytometry. Images are representative of at least three independent experiments. [file Image_1.tif]
