# Supplementary material for: The Shigella Type III Secretion Effector IpaH4.5 Targets NLRP3 to Activate Inflammasome Signaling
Source: Front Cell Infect Microbiol. 2020 Sep 30;10:511798. doi: 10.3389/fcimb.2020.511798 (PMC7561375; doi:10.3389/fcimb.2020.511798)
Supplement: Supplementary Table 1 — Strains and plasmids used for this study. [file Table_1.doc]

**Supplementary Table S1.** Strains and plasmids used for this study

| ***Strain, plasmid*** | ***Characteristic*** | ***Reference or source*** |
| --- | --- | --- |
| **Strains** |  |  |
| *S. flexneri* 301 | *S. flexneri* serotype 2a, WT | *Zirui Zheng et al (2016)* |
| Δ*IpaH4.5* mutant | *S. flexneri IpaH4.5* gene deletion mutant | *Zirui Zheng et al (2016)* |
| Δ*IpaH4.5*/*IpaH4.5*  △IpaH4.5/C379A | *S. flexneri* *IpaH4.5* deletion mutant complemented with *IpaH4.5*  S.flexneri IpaH4.5 deletion mutant complemented with IpaH4.5 C379A | *Zirui Zheng et al (2016)*  *Zirui Zheng et al (2016)* |
| *E. coli* DH5α/ BL21 | Employed for DNA amplification and recombinant protein expression | TINGKE biological technology |
| **Plasmids** |  |  |
| Flag-Vector | pcDNA3.1-Flag | Invitrogen Life Technologies |
| Flag-NLRP3 | NLRP3 sequence cloned into pcDNA3.1-Flag | Preservation in the laboratory |
| Flag-IpaH4.5 | IpaH4.5 sequence cloned into pcDNA3.1-Flag | Preservation in the laboratory |
| Flag-IPAF  Flag-ASC | IPAF sequence cloned into pcDNA3.1-Flag  ASC sequence cloned into pcDNA3.1-Flag | Preservation in the laboratory  Preservation in the laboratory |
| Myc-Vector | pCMV-Myc | Invitrogen Life Technologies |
| Myc-IpaH4.5 | IpaH4.5 sequence cloned into pCMV-Myc | Preservation in the laboratory |
| Myc-IpaH4.5 C379A | IpaH4.5 C379A sequence cloned into pCMV-Myc | Preservation in the laboratory |
| Myc-IpaH7.8 | IpaH7.8 sequence cloned into pCMV-Myc | Preservation in the laboratory |
| GST-Vector | pGEX4T-1 vector expressing GST for pull down | Amersham Biosciences Biotech |
| GST-IpaH4.5 | Vector expressing GST-tagged IpaH4.5 for pull down | Preservation in the laboratory |
| HA-Ub | Ub sequence cloned into pCMV-HA | Preservation in the laboratory |
| HA-Ub 48 | All sites are mutated except lysine 48 | Preservation in the laboratory |
| HA-Ub 63 | All sites are mutated except lysine 63 | Preservation in the laboratory |
